# 20 years later: unravelling the genomic success of New Zealand’s home-grown AK3 community-associated methicillin-resistant Staphylococcus aureus

**DOI:** 10.1099/mgen.0.001452

**Published:** 2025-07-25

**Authors:** Rhys T. White, Sarah Bakker, Maxim Bloomfield, Megan Burton, Juliet Elvy, Alexandra Eustace, Nigel P. French, Jenny Grant, Sabrina S. Greening, Alex Grinberg, Chad Harland, Samantha Hutton, Ali Karkaba, Jesse Martin, Brya Matthews, Hilary Miller, Christina Straub, Claire Tarring, William Taylor, James Ussher, Charles Velasco, Emma M. Voss, Kristin Dyet

**Affiliations:** 1 Institute of Environmental Science and Research, Health Security, Porirua 5022, New Zealand; 2 Department of Microbiology and Molecular Pathology, Awanui Labs Wellington, Wellington 6021, New Zealand; 3 Infection Services, Capital, Coast & Hutt Valley, Te Whatu Ora/Health New Zealand, Wellington 6021, New Zealand; 4 Department of Microbiology and Molecular Pathology, Awanui Labs Dunedin, Dunedin 9016, New Zealand; 5 Tāwharau Ora | School of Veterinary Science, Massey University, Palmerston North 4474, New Zealand; 6 Wildlife Futures Program, School of Veterinary Medicine, New Bolton Center, University of Pennsylvania, Kennett Square 19348, USA; 7 Research and Development, Livestock Improvement Corporation, Newstead 3286, New Zealand; 8 Cognosco, Anexa Veterinary Services, Morrinsville 3300, New Zealand; 9 Department of Molecular Medicine and Pathology, University of Auckland, Auckland 1142, New Zealand; 10 Centre for Microbiology and Environmental System Science, University of Vienna, Vienna, Austria; 11 Centre for Microbiology and Environmental System Science, University of Otago, Dunedin 9016, New Zealand

**Keywords:** antibiotic resistance, genomic surveillance, infection control, outbreak detection, phylogenetic analysis

## Abstract

Methicillin-resistant *Staphylococcus aureus* (MRSA) represents a significant public health challenge. In New Zealand, the community-associated MRSA sequence type (ST)5, carrying the staphylococcal cassette chromosome *mec* (SCC*mec*) type IV genetic element (which confers methicillin resistance), has been predominant since its detection in 2005. Known informally as the AK3 strain, it also exhibits resistance to fusidic acid. Here, we investigated the genomic evolution of the AK3 strain by analysing 397 genomes, comprising 361 MRSA and 36 closely related methicillin-susceptible *S. aureus* (MSSA) genomes, including 285 recently sequenced isolates from New Zealand spanning 2020 (*n*=30), 2021 (*n*=77), 2022 (*n*=88), 2023 (*n*=73) and 2024 (*n*=17). Phylogenetic analysis revealed that the AK3 strain evolved through stepwise acquisition of mobile genetic elements, with an MSSA ancestor likely introduced to New Zealand in the late 1970s. The lineage first acquired a SaPITokyo12571-like pathogenicity island, which contains the staphylococcal enterotoxin C bovine variant (*sec*-bov) and an enterotoxin-like protein (*sel*), between 1984 and 1991. This was followed by the integration of SCC*mec* type IV and adjacent fusidic acid resistance operon between 1997 and 2000. This timing coincides with increased community fusidic acid use in New Zealand. The AK3 strain then diversified into three major clades, spreading throughout New Zealand and Australia, with sporadic detection in European countries and Samoa. Our findings demonstrate how the sequential acquisition of mobile genetic elements, combined with antibiotic selection pressure, likely contributed to the successful emergence of AK3 and its spread in the South Pacific region.

**Impact Statement**

This research represents a substantial advancement in understanding the evolutionary history of the fusidic acid-resistant ST5-MRSA-SCC*mec*-IV (informally known as the AK3 strain). The AK3 strain is a major contributor to methicillin-resistant *Staphylococcus aureus* (MRSA) infections in New Zealand. By analysing 397 genomes, including 285 recently sequenced under national surveillance efforts, we identified key genetic factors and evolutionary events driving the success of AK3-related strains. We extended the temporal and spatial scope of AK3 research, quantified its national and cross-hospital spread and highlighted mobile genetic elements shaping its persistence and resistance. By incorporating sampling from bovine sources, we also uncovered potential non-clinical reservoirs, emphasizing a critical One Health context. These findings address key gaps in prior studies, providing actionable insights for genomic surveillance, infection control and the development of equitable, culturally responsive public health strategies to combat MRSA.

## Data Summary

The study sequences are available in the National Center for Biotechnology Information (NCBI) under BioProject accession numbers PRJNA1029301, PRJNA1046639, PRJNA1090129 and PRJNA1144171. The raw sequence read data generated in this study have been deposited to the NCBI sequence read archive (SRA) (https://www.ncbi.nlm.nih.gov/sra), and accession numbers are listed in Tables S1 and S2, available in the online version of this article. The complete assembly for strain sa230905_barcode06 has been deposited to GenBank under the accession number CP168012. The software used to analyse raw sequence reads for polymorphism discovery and whole-genome sequencing-based phylogenetic reconstruction are available as described in Methods. The authors confirm that all supporting data protocols have been provided in the article or supplementary data files.

## Introduction

Methicillin-resistant *Staphylococcus aureus* (MRSA) presents a serious public health challenge due to the associated antibiotic resistance, making infections substantially harder to treat than those caused by methicillin-susceptible *S. aureus* (MSSA) strains [1]. In 2005, a fusidic acid-resistant MRSA sequence type (ST)5 strain carrying staphylococcal cassette chromosome *mec* (SCC*mec*)-IV, informally known as the AK3 strain [2], was first identified in New Zealand (known as Aotearoa in the Māori language) [3]. AK3 represents a significant shift in the epidemiology of community-associated MRSA (CA-MRSA). AK3 became the predominant CA-MRSA strain by 2009, accounting for 25.8% of all MRSA isolations, surpassing the previously dominant Western Samoan phage pattern (WSPP) strain [4,5].

Before the emergence of AK3, the ST30 WSPP clone was a predominant cause of MRSA infections across New Zealand from the 1980s to the early 2000s [6]. ST30 WSPP harbours SCC*mec*-IV and the Panton–Valentine leukocidin (PVL) toxin [7] (encoded by two co-transcribed genes, *lukS-PV* and *lukF-PV* [8]), which are associated with severe skin infections. However, surveillance data from 2006 revealed AK3 as an emerging cause of CA-MRSA infections in New Zealand [9]. AK3 prevalence significantly increased from 3.8% (579 cases) in 2006 to 25.8 % (693 cases) in 2009, becoming the predominant CA-MRSA strain in New Zealand [5,10]. Nonetheless, the rise of AK3 presents an epidemiological puzzle.

Unlike many successful MRSA strains, AK3 was reported to be negative for the genes encoding the PVL toxin based on PCR results [2]. The role of the PVL toxin in *S. aureus* infections remains controversial. While PVL-positive strains have been linked to distinct clinical characteristics, like younger patient age and community acquisition [11], the direct impact on infection severity is not fully understood. Studies have shown that PVL-negative strains can be as virulent as PVL-positive strains in certain models [12,14]. Therefore, the success of AK3 may reflect alternative virulence mechanisms or its ability to disseminate effectively within specific ecological niches.

AK3 likely evolved from a local New Zealand MSSA ST5 strain following the acquisition of SCC*mec*-IV [15]. This element carries the *mec*A gene (encoding the penicillin‐binding protein PBP2a) and the adjacent *fus*C gene, which confers resistance to fusidic acid – an antibiotic that has been widely used in topical formulations [16,17]. This genomic arrangement is particularly significant given the dramatic increase in fusidic acid use in New Zealand: community dispensing rates of topical fusidic acid rose from <0.5 per 1,000 population per month before 1999 to >2 per 1,000 by January 2001 [18]. Community dispensing rates for topical fusidic acid are highest in New Zealand’s Northern region and among Māori and Pacific peoples [19]. This antibiotic pressure likely drove the emergence of AK3 and subsequent dissemination [2,17, 19], particularly affecting vulnerable populations. Children under 5 years old show the highest period-prevalence rates of MRSA infections, while Māori, Pacific Island and other minority communities are disproportionately affected [20,21]. Beyond human infections, ST5 strains circulate in livestock [22,24], poultry [25] and companion animals [26], exhibiting host-specific adaptations and antimicrobial resistance (AMR). Notably, a human-to-poultry host jump has been estimated to have occurred between the 1940s and 1980s [25]. The presence of ST5 in diverse reservoirs suggests a complex ecological network facilitating persistence and spread.

This study examines the recent evolutionary history of the AK3 (ST5-MRSA-SCC*mec*-IV) strain in New Zealand, identifying the genomic factors that contributed to its rapid expansion and dominance. By addressing gaps in previous research, including the transmission dynamics, genomic drivers and potential non-human reservoirs of AK3-related strains, this work provides a foundation for more targeted public health strategies to mitigate CA-MRSA transmission and its associated health impacts.

## Methods

### Sample collection/study design

In New Zealand, the incidence of invasive and non-invasive *S. aureus* infections is higher than in many other high-income countries, with the highest rates observed in Māori and Pacific peoples [20,21]. The 2021–2023 *S*. *aureus* bacteraemia (SAB) surveillance programme [led by the Institute of Environmental Science and Research (ESR)] was conducted on behalf of the Ministry of Health New Zealand (Māori: Manatū Hauora) to document rates of AMR, to better characterize the molecular epidemiology of isolates causing SAB, to examine variations in mortality rates and to examine variations in SAB rates according to place of onset, patient age, ethnicity, socioeconomic deprivation, risk factors and comorbidities.

All New Zealand diagnostic laboratories were requested to refer (to ESR) *S. aureus* isolates from blood culture samples from 1 January 2021 to 31 December 2023 for further characterization. Duplicate isolates from the same patient, which were cultured within 14 days of the initial sample, were not included, unless an isolate of a different staphylococcal protein A gene (*spa*) type was identified. Isolates were characterized using a real-time PCR that detected the methicillin resistance genes *mecA* and *mecC* and *lukS*-PV for the detection of PVL [27]. Isolates were also characterized using *spa* typing [28] and, if needed, PFGE [29]. All MRSA isolates, as defined by the presence of functional *mec*A or *mec*C genes, underwent whole-genome sequencing (WGS) using Illumina-based sequencing technology (described below). Non-blood culture isolates were acquired by referral from diagnostic laboratories as part of routine hospital outbreak investigation work, performed at ESR by the national Antimicrobial Resistance Laboratory.

### Illumina library construction and next-generation sequencing

As part of the ESR national staphylococcal surveillance surveys, *Staphylococcus* cultures are plated on blood agar and incubated at 35 °C with 5% carbon dioxide (CO_2_) for 18 h. Following incubation, the cultures are visually examined for viability and purity. A subculture of a single colony pick is then transferred to another blood agar plate and incubated at the same conditions for 18 h. The heat-killed cell suspensions from an overnight culture of a single colony pick were extracted using the chemagic™ 360 instrument (PerkinElmer Inc., Waltham, MA, USA). The DNA library was created using the PlexWell Library Preparation kit (seqWell™, Boston, MA, USA) and sequenced as 2×151 bp paired-end reads on the NextSeq 550 platform using V2.5 chemistry (Illumina Inc., San Diego, CA, USA) at ESR (Kenepuru, Porirua, New Zealand).

### Quality control and *de novo* assembly of the Illumina sequence read data

Raw reads were checked for quality using FastQC v0.11.9 (http://www.bioinformatics.babraham.ac.uk/projects/fastqc/, accessed on 12 December 2024). To perform taxonomic profiling and detect *S. aureus* in the raw Illumina sequence data, we used Kraken v2.1.3 [30] with default parameters (in paired-end read mode) and an NCBI Reference Sequence (RefSeq) database [31], Standard (https://benlangmead.github.io/aws-indexes/k2, accessed on 12 December 2024). This database contained references for archaea, bacteria, human, viruses, plasmids and the ‘UniVec core’ subset of the UniVec database (a database of vector, adaptor, linker and primer sequences). Raw Illumina sequence reads were *de novo* assembled using Shovill v1.1.0 (https://github.com/tseemann/shovill, accessed on 12 December 2024), which utilizes the following: Seqtk v1.3-r106 (https://github.com/lh3/seqtk, accessed on 12 December 2024), Trimmomatic v0.36 [32], Lighter v1.1.2 [33], FLASH v1.2.11 [34], SKESA v2.4.0 [35,36], Samclip v0.4.0 (https://github.com/tseemann/samclip, 12 December 2024), SAMtools v1.16.1 [37], the Burrows-Wheeler Aligner (BWA) v0.7.17 [38] and Pilon v1.24 [39]. Shovill was used with parameters set to (i) estimate the genome size to 2.8 Mb. (ii) remove contiguous sequences (contigs) with a sequence coverage below 20-fold and (iii) enable single-cell mode. Assembly metrics were assessed using QUAST v5.0.2 [40].

### Sampling and extraction of DNA for nanopore sequencing

Isolates for nanopore sequencing were collected from Wellington Regional Hospital and comprise two sets of *S. aureus* samples [41,42]. These isolates were sequenced using Oxford Nanopore Technologies (ONT) as part of a local, prospective decentralized WGS programme. ONT was used instead of Illumina to support rapid, real-time genomic surveillance and timely infection prevention and control responses during suspected transmission events [41,45]. The first set consists of four *S. aureus* cases collected in February (*n*=2), March (*n*=1) and April (*n*=1) 2022. The second set consists of six *S. aureus* cases collected between July and the end of December 2023. In both sets of samples, the samples were promptly processed upon arrival at Awanui Labs (Wellington, New Zealand). Two samples are from blood culture isolates, which were obtained from positive patient samples (BacT/ALERT® system, bioMerieux, Marcy-l’Etoile, France). Positive bottles were subcultured onto 5% sheep blood agar at 37 °C for 24 h, with further subcultures taken for the purposes of sequencing. The remaining eight of these ten clonal complex (CC)5 samples were obtained from eye or screening swabs using the standard bacterial swab technique in Amies transport medium. For MRSA screening, swabs were collected from various sites, including the nose, axilla, umbilicus, groin and any open wounds. These swabs underwent culture for 18–24 h on the CHROMagar™ MRSA (St-Denis, France) and enrichment in 7% salt broth for 18 h, with subculture onto CHROMagar™ MRSA for a further 18–24 h. CHROMagar MRSA is a selective and differential agar, which inhibits the growth of MSSA and most other bacteria. Suspicious MRSA isolates (Mauve colonies) were tested phenotypically for confirmation against cefoxitin and oxacillin and susceptibility to multiple agents using the Vitek II instrument (bioMerieux) and the AST-P656 card, according to the European Committee on Antimicrobial Susceptibility Testing (EUCAST) methods [46].

Gram-positive DNA extraction involves suspending a 10 µl loop of bacteria in 300 µl of PBS and freezing at −20 °C for at least 24 h. The thawed solution is vortexed for 1 min with a small quantity of 0.1 mm zirconia/silica beads (dnature, Gisborne, New Zealand; SKU 11079101Z) and then centrifuged at ~12,000 ***g*** for 30 s at room temperature, with the supernatant used for sequencing without further clean-up. For isolates sequenced between February and April 2022, libraries were constructed using 50 ng of genomic DNA with the ONT rapid barcoding kit 96 (SQK-RBK110-96, Oxford, UK) as per the manufacturer’s instructions. Subsequently, the entire library was loaded onto an R9.4 flow cell (FLO-MIN106) and run on a MinION™ device for ~20–40 h (using MinKNOW v22.10.10). After March 2023, libraries were created using 50 to 100 ng of genomic DNA, prepared using the rapid barcoding kit 96 (SQK-RBK114-96, Oxford) and sequenced on an R10.4.1 flow cell (FLO-MIN114) with MinKNOW v23.04.5. Further details of nanopore basecalling and nanopore read quality control are available in the Supplementary Materials.

### Dataset curation

In addition to the 449 *S*. *aureus* CC5 genomes sequenced in this study (Table S1), an additional 67 publicly available CC5 genomes from New Zealand studies [22,24,26, 47] were added to this dataset (Table S2). Further details of *de novo* assembly are available in the Supplementary Materials. Another 6,551 publicly available *S. aureus* CC5 genome assemblies were downloaded using the PathogenWatch platform (https://pathogen.watch/, accessed on 08 October 2024). Finally, four complete CC5 genome assemblies were downloaded from the NCBI Assembly database: Mu50 (human, pus, Japan, 1997, GenBank: BA000017), N315 (human, pharyngeal smear, Japan, 1982; GenBank: BA000018), JH1 (human, blood, USA, 2000, GenBank: CP000736) and NZAK3 (human, skin, New Zealand, 2005, GenBank: LT009690).

### *In silico* genotyping of the *S. aureus* genome sequence data

*In silico* multi-locus sequence typing (MLST) was done using MLST v2.23.0 (https://github.com/tseemann/mlst, accessed on 12 December 2024) with default settings to query the assemblies against the *S. aureus* typing database [48] hosted on PubMLST hosted on BIGSdb v1.47.0 [49] (local database updated 19 March 2024). The *spa* types were identified using spaTyper v1.0.0 [50], a tool that utilized the spa typing website (*http://www.spaserver.ridom.de/*, accessed on 16 December 2024) developed by Ridom GmbH and maintained by SeqNet.org (http://www.SeqNet.org/, accessed on 16 December 2024). Virulence genes, acquired antibiotic resistance genes and mutations conferring resistance to antibiotics were identified using AMRfinderplus v3.12.8 with database v2024-01-31.1 [51].

### Assembly-based variant detection and ST5 phylogenetic analyses

A total of 7,071 *S*. *aureus* CC5 genome assemblies were aligned to create a core-genome alignment using Parsnp v1.7.4 [52], with the reference being the earliest collected isolate (chromosome of MRSA ST5 strain Mu50), to identify single-nucleotide variants (SNVs). Resulting SNV alignments were used to reconstruct phylogenies. RaxML v8.2.12 [53] built phylogenetic trees using the maximum-likelihood method with general time-reversible (GTR)-GAMMA correction (optimizing 20 distinct, randomized maximum-parsimony trees before adding 1,000 bootstrap replicates). The phylogenetic trees were visualized using FigTree v1.4.4 (http://tree.bio.ed.ac.uk/software/figtree/, accessed 05 November 2024) and EvolView v2 [54,55].

### Genome annotation for sa230905_barcode06

Due to the unavailability of a complete genome representative of a closely related outgroup to the MRSA AK3 lineage, we selected sa230905_barcode06 as the reference genome. sa230905_barcode06 is a clinical isolate collected from a neonatal eye swab at the Wellington Regional Hospital neonatal intensive care unit in August 2023. The FASTQ files outputted from the basecalling were corrected with single-read error correction using the haplotype-aware error correction (HERRO) algorithm (using the ‘dorado correct’ function using model-v1, Dorado v0.7.3) [56]. HERRO-corrected reads underwent *de novo* assembly using Flye v2.9.4 [57,58] using a genome size estimate of 2.8 Mb and three polishing iterations [59]. The assembly representing strain sa230905_barcode06 was annotated using Prokka v1.14.6 [60]. Prophage regions were identified using PHASTEST v3.0 [61,63] and then annotated using Pharokka v1.5.1 [64]. Mobile genetic elements were identified using IslandViewer 4 [65] and ISsaga v2.0 [66] (ISfinder platform [67]), followed by manual curation using Artemis v18.2.0 [68].

### High-resolution cluster phylogeny

Our dataset for the AK3 lineage consisted of 397 genomes. The SPANDx v4.0.4 pipeline [69] was utilized for identifying genetic variants through a read-mapping method, as described previously [42]. Briefly, Illumina short reads or simulated short reads from assembled (nanopore) genomes (see SPANDx manual) were mapped to the complete sa230905_barcode06 chromosome (GenBank: CP168012). SNVs within regions of high-density clusters (≥3 SNVs found within a 10 bp window) and predicted recombination sites (identified using Gubbins v3.3.5 [70]) were removed from the core-genome alignment. Sites were excluded if an SNV was called in regions with less than half or greater than threefold the average genome coverage on a genome-by-genome basis. For this analysis, the core genome was defined as the set of genomic regions (in 100 bp windows) that had ≥95% sequence coverage in all genomes, as calculated using the BEDTools v2.28.0 [71] coverageBed module within the SPANDx pipeline [69].

A maximum-parsimony tree was reconstructed from the orthologous biallelic core-genome SNV alignment using the heuristic search feature of PAUP v4.0a [72]. The core-genome SNV alignment was independently run through jModelTest v2.1.10 [73], to identify the best-fit evolutionary model using 12 candidate models from 3 substitution schemes with the base tree for likelihood calculations optimized for maximum likelihood. jModelTest included models with equal/unequal base frequencies (+F), with/without a proportion of invariable sites (+I) and with/without rate variation among sites (+G) (four rate categories). Maximum-likelihood phylogenetic trees were generated from the orthologous biallelic core-genome SNV alignments using RAxML (GTR correction) as described above. The resulting phylogenetic trees were visualized using FigTree and EvolView. Three clades (AK3 Clades 1, 2.1 and 2.2) were defined based on the topology of the maximum-parsimony phylogeny constructed from non-recombinant core-genome SNVs. To further identify robust phylogenetic groupings within the AK3 lineage, we used the core-genome SNV alignment as input into rhierBAPS v1.0.1 [74], an R v4.3.2 [75] implementation of the hierarchical Bayesian analysis of population structure (BAPS) algorithm [76]. We specified one level of clustering and allowed up to ten initial clusters. The resulting BAPS groupings were congruent with the three visually defined clades (AK3 Clades 1, 2.1 and 2.2) from the maximum parsimony.

### Divergence estimates of the *S. aureus* ST5 AK3 lineage

To calibrate the phylogeny, we used tip-dating methods using TempEst v1.5.3 [77] and Bayesian ancestral state reconstruction using BEAST2 v2.7.7 [78,79]. In the Bayesian method, we initially assessed whether the strict or optimized relaxed uncorrelated clock model is more suitable for our dataset. The initial models were created using tip dates, a GTR substitution model and a coalescent prior with a constant population. Both models were tested with the nested sampling Bayesian computation algorithm v1.1.0 within the BEAST2 package with a particle count of 32, sub-chain length of 5,000 and epsilon of 1.0×10^−12^. This analysis provides evidence in favour of the uncorrelated relaxed clock model. The Bayesian skyline, coalescent constant and exponential growth population size change models were evaluated for the optimized relaxed uncorrelated clock model. The Gamma Site Model Category Count was set to four, and the GTR substitution model rates determined from jModelTest were included (i.e. rate AC=0.99, AG=3.84, AT=0.74, CG=0.21, CT=3.84 and GT=1.00). The initial clock rate was set to 2.42×10^−4^ substitutions per site per year (estimated from the root-to-tip regression analysis in TempEst) with a uniform distribution and an upper bound of 0.1. All other priors were left as default. After identifying the most suitable tree model, three separate Markov chain Monte Carlo runs were performed for 100 million generations for each analysis. Trees were collected every 1,000 generations, yielding 3 sets of 100,000 trees for each model test. To evaluate the metrics, all BEAST2 executions were loaded into Tracer v1.7.2 (http://github.com/beast-dev/tracer/, accessed on 14 November 2024). LogCombiner v2.7.7 (part of the BEAST2 package) subsequently combined the replicated analyses for each model with a 10% burn-in to evaluate convergence. TreeAnnotator v2.7.7 (part of the BEAST2 package) produced maximum clade credibility trees from each run (derived from 270,000 trees), annotating median values with a posterior probability limit set at 0.5. The phylogenetic trees produced were visualized in FigTree and EvolView.

## Results

### Overall *S. aureus* CC5 population expands the representation of New Zealand AK3 sub-lineage

For this analysis, we analysed 7,071 genomes in total (Table S3). These 7,071 *S*. *aureus* CC5 genomes include 449 sequenced in this study (Table S1), 67 publicly available from New Zealand studies (Table S2), 6,551 publicly available assemblies hosted on the PathogenWatch platform and 4 complete assemblies from the NCBI Assembly database. The chromosome of Mu50 was used as a reference to call SNVs. The core-genome alignment captured ~15.3% of the reference chromosome (439,853 bp of the 2,878,529 bp Mu50 chromosome), yielding 32,653 SNVs for phylogenetic inference.

**Fig. 1.**
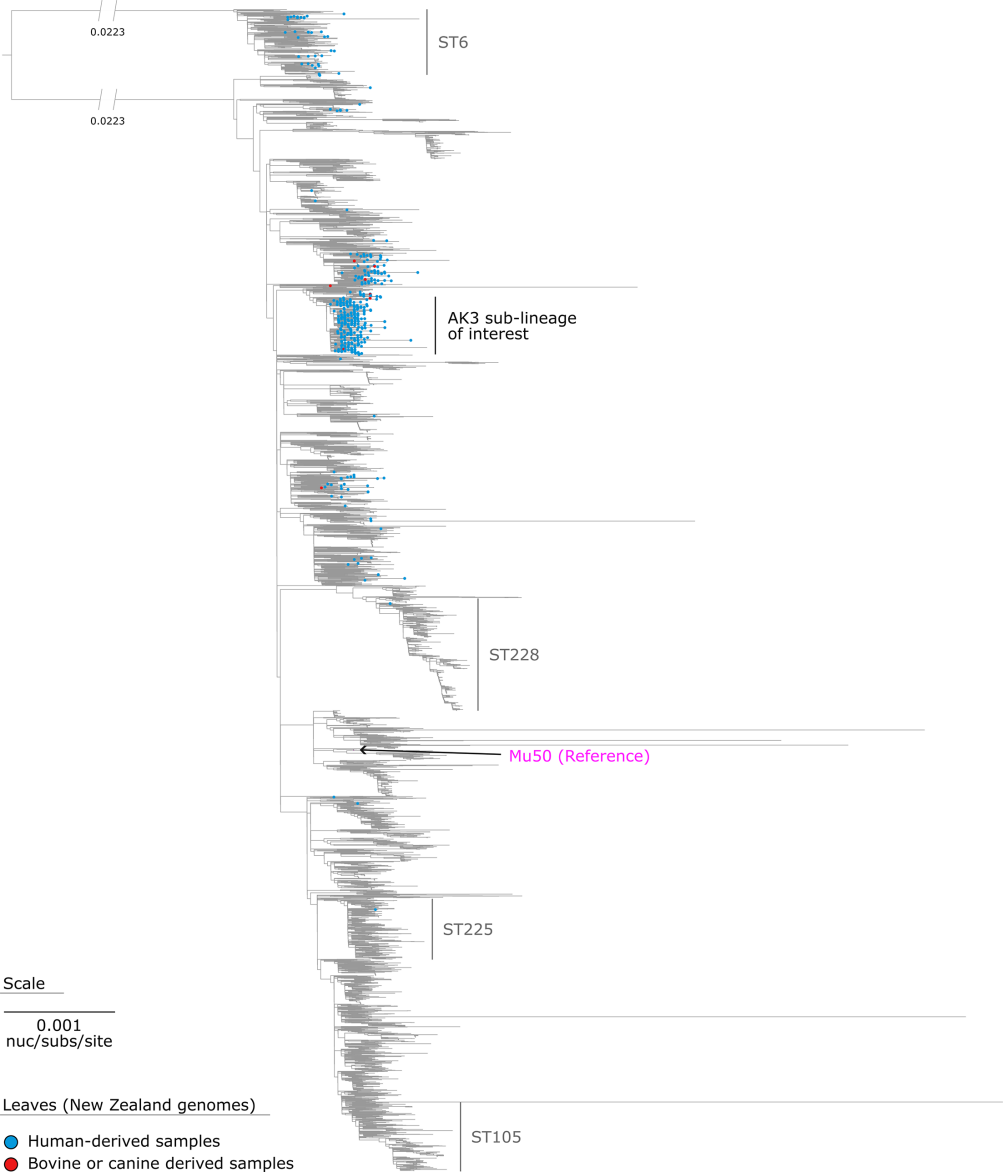
Maximum-likelihood phylogeny of *S. aureus* CC5. The phylogeny was inferred from 32,653 core-genome SNVs from 7,071 assembled genomes. SNVs were derived from a core-genome alignment of 439,853 bp and are called against the 2,878,529 bp chromosome of Mu50 (GenBank: BA000017). An ST comprised of >100 genomes is labelled. The maximum-likelihood phylogeny was outgroup-rooted using the *S. aureus* ST6 lineage.

The phylogenetic analysis revealed a distinct population structure within CC5, with several major STs forming monophyletic clades (Fig. 1). Within CC5, we identified multiple distinct clades, with the most prominent (other than ST5) being ST228, ST225 and ST105, each containing more than 100 genomes. The sub-lineage (commonly referred to as MRSA ST5 AK3) of epidemiological interest to New Zealand contains 328 human-derived *S. aureus* CC5 isolates from New Zealand. There is minimal representation of bovine-derived isolates (*n*=3) in the AK3 sub-lineage of interest. Notably, the reference genome Mu50 is located within the broader CC5 population structure and is distinct from the AK3 sub-lineage of interest.

### A high-quality methicillin-susceptible reference genome for *S. aureus* ST5 AK3 lineage

WGS of the sa230905_barcode06 genome, used as the reference genome in this study, generated 79,071 single-ended reads. After filtering out reads shorter than 10,000 bp and those with a mean Q-score below 10 (indicating 90% accuracy per base, or a 1 in 10 chance of an error in the base call), and applying HERRO correction implemented in Dorado, 7,748 high-quality reads remained. These reads totalled 117,422,744 bases, with a median length of 13,490 bp and a read N50 length of 15,358 bp (Table 1). *De novo* assembly of sa230905_barcode06 revealed a circular chromosome of 2,807,155 bp with 32.86% GC content, with a median sequencing depth of 42×.

**Table 1.** Genomic characteristics of *S. aureus* strain sa230905_barcode06

Attribute	Value
*Metadata*	
Collection date	August 2023
Source	Human
Sample type	Eye swab
*Raw data*	
Median read length (base pairs)	2,424
Median read quality	24.2
Number of reads	79,071
Read length N50	7,584
Total number of bases	326,004,082
*Post HERRO error correction*	
Median read length (base pairs)	13,490
Number of reads	7,748
Read length N50	15,358
Total number of bases	117,422,744
*De novo assembly*	
Average sequencing depth	42×
No. contigs	1
Total length (base pairs)	2,807,155
GC (%)	32.86
*Typing*	
MLST	ST5
*spa* type	t1062
Predicted SCC*mec* element	None
No. of prophage elements	3
Antimicrobial resistance genes on the chromosome	*tet*(38); *bla*I; *bla*Z; *fos*B
Virulence genes on the chromosome	*ads*A; *spa*; *cap*A; *cap*8B; *cap*8C; *cap*8D; *cap*8E; *cap*8F; *cap*8G; *cap*8L; *cap*8M; *cap*N; *cap*8O; *cap*8P; *isd*I; *esx*A; *esa*A; *ess*A; *esa*B; *ess*B; *ess*C; *esx*C; *esx*B; *esa*E; *esx*D; *esa*D; *esa*G; *esa*G; *esa*G; *esa*G; *esa*G; *esa*G; *geh*; *set*16; *set*17; *set*18; *set*19; *set*20; *set*22; *set*23; *set*24; *set*25; *set*26; *sdr*C; *sdr*D; *sdr*E; *sec*; *sel*l; *clf*A; *ssp*C; *ssp*B; *ssp*A; *isd*B; *isd*A; *isd*C; *isd*D; *isd*E; *isd*F; *srt*B; *isd*G; *hly*/*hla*; *ebp*; *har*A; *luk*F-PV; *eap*/*map*; *scn*; *chp*; *sak*; *sea*; *hlb*; *hld*; *hys*A; *sbi*; *hlg*A; *hlg*C; *hlg*B; *fnb*B; *fnb*A; *clf*B; *aur*; *ica*R; *ica*A; *ica*D; *ica*B; *ica*C; *lip*
*Data deposition*	
BioProject ID	PRJNA1144171
BioSample ID	SAMN43040015
SRA accession number	SRR30149079
GenBank accession number	CP168012

No SCC*mec*, which can carry the *mec*A gene, was detected in the sa230905_barcode06 chromosome. No plasmid was detected in either the initial or HERRO-corrected sequence data. The sa230905_barcode06 chromosome includes notable features such as the *spa* gene (typed as t1062) encoding immunoglobulin G binding protein A, an Ess/type VII secretion system, two major pathogenicity islands, a SaPITokyo12571-like element (GenBank: AB860417) and three prophages (Table S4). The sa230905_barcode06 chromosome harbours four AMR genes (Table 1). The *tet*(38) gene encodes a tetracycline efflux pump that can confer resistance to tetracycline. The *bla*I and *bla*Z genes encode regulatory and beta-lactamase proteins, respectively, which may confer resistance to beta-lactam antibiotics. The *fos*B gene encodes a fosfomycin resistance protein (FosB1/FosB3 family fosfomycin resistance bacillithiol transferase) that can inactivate the antibiotic fosfomycin. Additionally, sa230905_barcode06 contains an extensive repertoire of virulence genes, including those associated with immune evasion (*ads*A, *spa*, *scn*, *chp* and *sak*), capsular polysaccharide biosynthesis (*cap*A-*cap*N), iron acquisition (*isd*A-*isd*G), toxin production (*hla*, *hlb*, *hld*, *sea*, *hys*A and *luk*F-PV), adhesion (*sdr*C-*sdr*E, *clf*A, *clf*B, *fnb*A and *fnb*B) and biofilm formation (*ica*A-*ica*D). Genes encoding components of the type VII secretion system (*esx*A-*esx*D and *esa*A-*esa*G) and other virulence factors (*ebp*, *geh*, *aur* and *lip*) were also identified (Table 1).

### Stepwise evolution of mobile genetic elements preceded the rapid expansion of the AK3 lineage

**Fig. 2.**
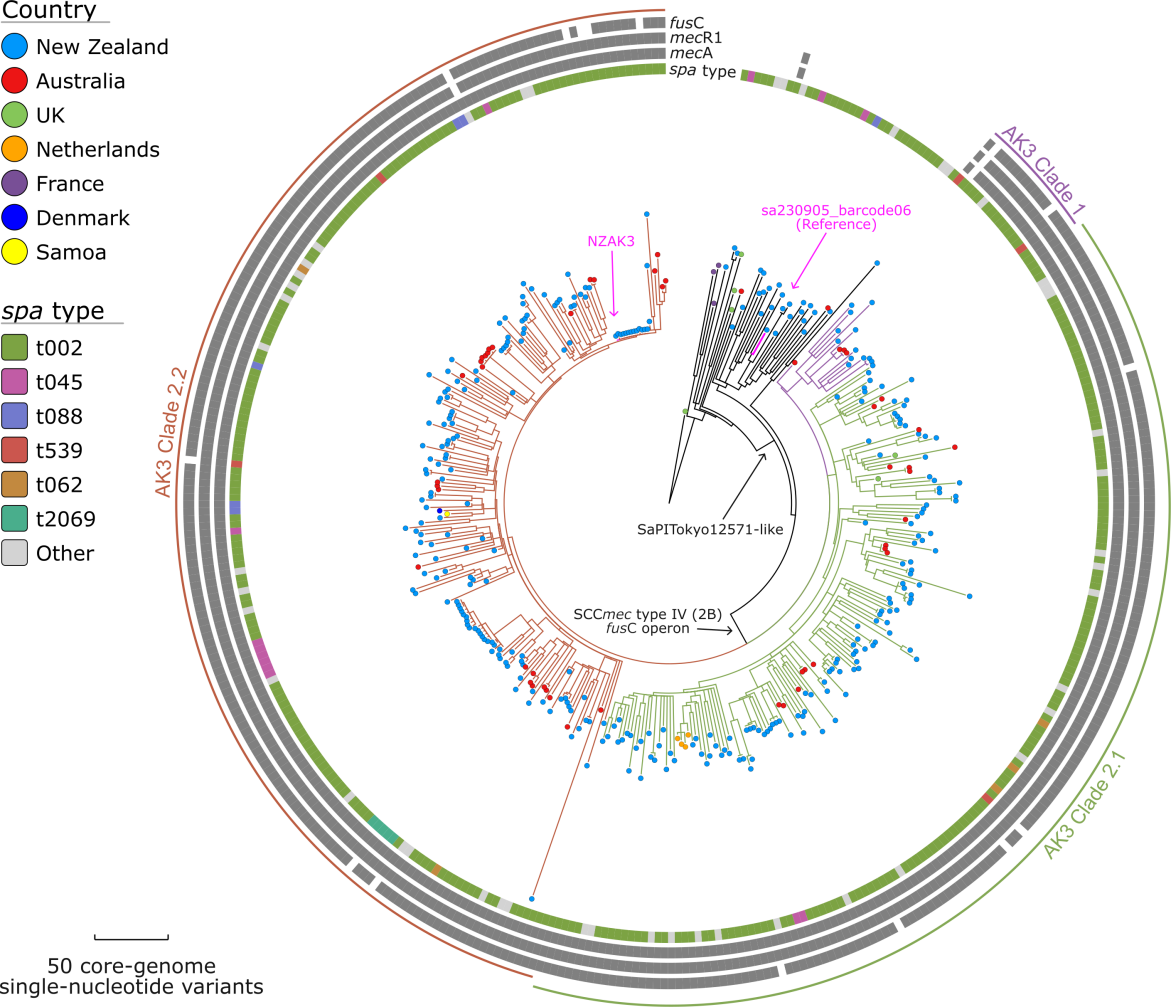
Maximum-parsimony phylogeny of the *S. aureus* AK3 lineage. The phylogeny was inferred from 10,062 non-recombinant orthologous biallelic core-genome SNVs from 397 genomes. SNVs were derived from a core-genome alignment of ~2,393,900 bp and were called against the chromosome of sa230905_barcode06 (GenBank: CP168012). The consistency index for the tree was 0.99. SNV density filtering in SPANDx (excluded regions with three or more SNVs in a 10 bp window). The phylogenetic tree was rooted according to the MSSA ST5 SRR13968194 outgroup (collected in the UK). Branch colours represent clades within the MRSA AK3 lineage: Clade 1 (purple), Clade 2.1 (green) and Clade 2.2 (red). For *mec*A, *mec*R1 and *fus*C, the grey plots indicated gene presence. Strain NZAK3 (GenBank: LT009690) was isolated in Auckland, New Zealand, in 2005 (from a patient with a skin and soft tissue infection) and represents the earliest known isolation of this fusidic acid-resistant AK3 ST5 MRSA clone globally.

Using the assembly-based CC5 phylogeny (Fig. 1), we refined our analysis to focus on the genomic data of strains within (*n*=361), as well as immediately outside (*n*=36), the AK3 lineage (Fig. 2). Of the 449 human-derived *S. aureus* genomes sequenced in this study, 285 (63.5%) belong to this lineage. These 285 genomes were compared with 112 publicly available *S. aureus* genomes that also fall in this lineage (Table S5). The 2,807,155 bp chromosome of sa230905_barcode06 (GenBank: CP168012) was used as a reference to call SNVs. The core-genome alignment captured~85.3% of the reference chromosome (~2,393,900 bp), yielding 10,062 non-recombinant orthologous biallelic core-genome SNVs for phylogenetic inference (see regions of recombination relative to the chromosome of sa230905_barcode06 in Table S6).

**Fig. 3.**
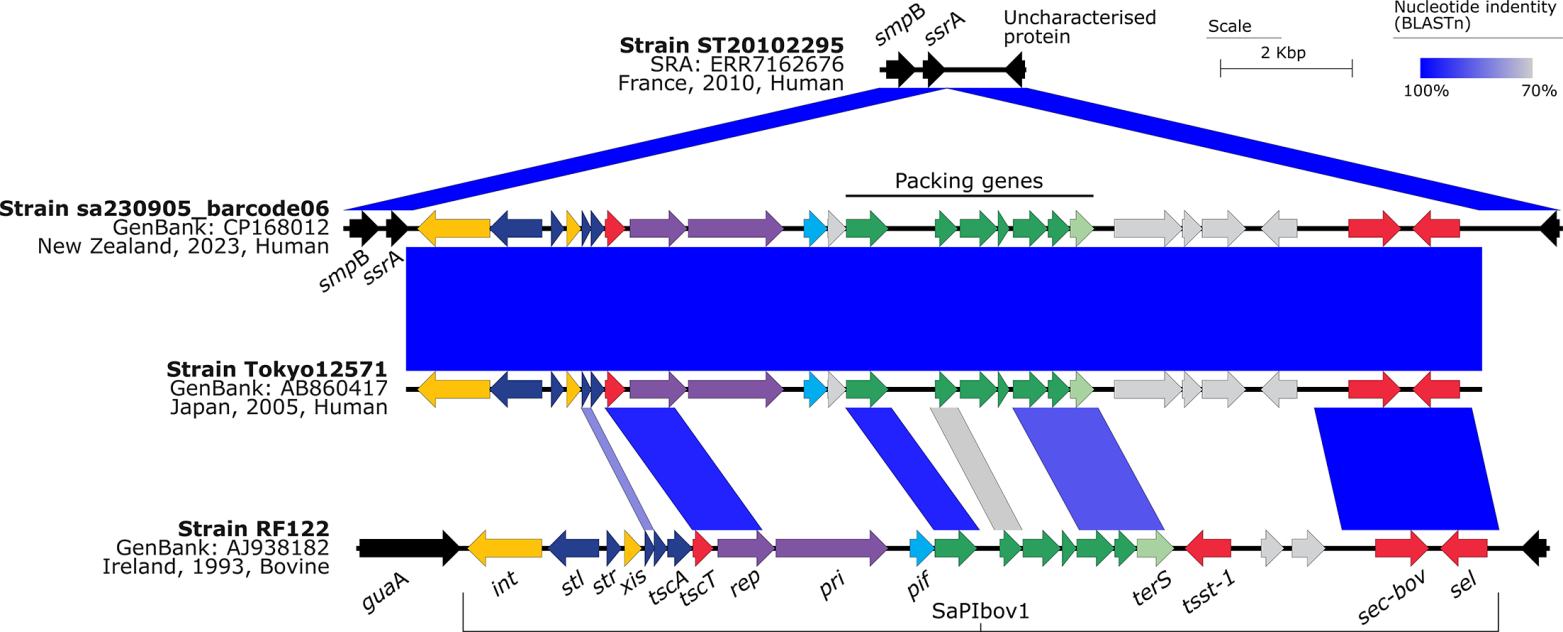
Major structural features and nucleotide pairwise comparisons of the *S. aureus* pathogenicity island SaPITokyo12571-like. Nucleotide comparisons between the MSSA ST5 t045 genome ST20102295 (SRA: ERR7162676), MSSA ST5 t1062 genome sa230905_barcode06 (GenBank: CP168012), *S. aureus* ST45 pathogenicity island SaPITokyo12571 (GenBank: AB860417) and MSSA ST151 t529 genome RF122 (GenBank: AJ938182). Blue shading indicates nucleotide identity between sequences according to blastn (70–100%). The direction of the arrows indicates the direction of transcription for ORFs. Key genomic regions are indicated: integrase (int) and excisionase (xis) in yellow, transcription regulators in dark blue, replication genes including the primase gene (*pri*), replication initiator gene (*rep*) in purple, encapsidation genes in green, terminase small subunit gene (*ter*S) in light green, superantigen and other accessory genes in red, *pif* (related to phage interference) in light blue, genes encoding hypothetical proteins in grey and flanking coding sequences in black. Image created using Easyfig v2.2.5 [94].

**Fig. 4.**
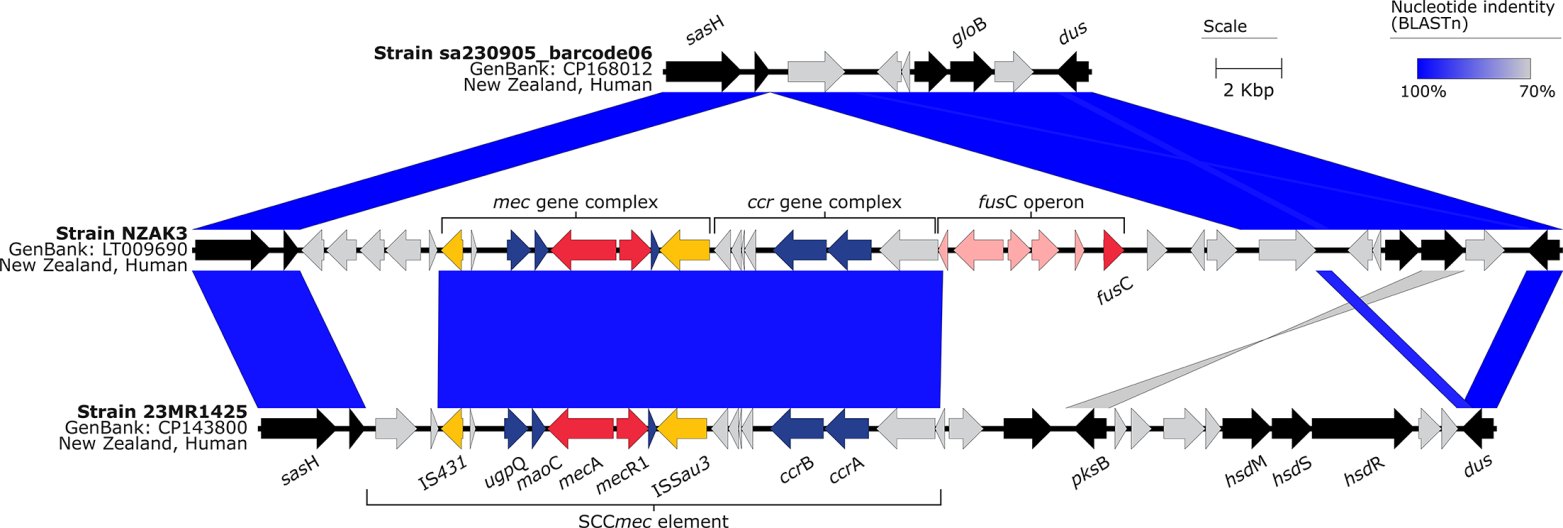
Major structural features and nucleotide pairwise comparisons of the staphylococcal cassette chromosome elements in *S. aureus* ST5 and ST97. Nucleotide comparisons between the MSSA ST5 t1062 genome sa230905_barcode06 (GenBank: CP168012), MRSA ST5 t002 genome NZAK3 (GenBank: LT009690) and MRSA ST97 t359 genome 23MR1425 (GenBank: CP143800). Blue shading indicates nucleotide identity between sequences according to blastn (70–100%). The direction of the arrows indicates the direction of transcription for ORFs. Key genomic regions are indicated: insertion sequences in yellow, genes conferring antibiotic resistance in red, genes present within SCC*mec* regions in blue, genes present within the fusidic acid operon in light red, genes encoding hypothetical proteins in grey and flanking coding sequences in black. Image created using Easyfig v2.2.5 [94].

The evolutionary history of the AK3 lineage demonstrates the stepwise acquisition of significant mobile genetic elements. The SaPITokyo12571-like element (Fig. 2) was acquired before the integration of the *fus*C operon and SCC*mec* type IV (2B) in a subsequent event. This sequential integration of mobile genetic elements (Figs 3, 4) appears to have preceded the expansion of what we now recognize as the contemporary AK3 lineage. Beyond the major integration events of the SaPITokyo12571-like element, and the *fus*C operon alongside the SCC*mec* type IV (2B), this analysis reveals clade-specific SNVs, in non-recombinant core-genome regions, which distinguish clades within the lineage (Tables2  3). These variants include synonymous and non-synonymous mutations, impacting genes with roles in essential cellular processes, such as replication, protein synthesis, transport and virulence.

**Table 2.** Clade-specific variants to the SaPITokyo12571-like element branch in the ancestor to the AK3 lineage

Position in sa230905_barcode06	ERR7162676 (outgroup)*	sa230905_barcode06 (branch)*	Change†	Impact	Codon	Gene	Product
408	**G**	*A*	Syn	Low	136	*dna*A	Chromosomal replication initiator protein DnaA
148,318	**A**	*C*	I=>L	Moderate	1456	*aus*A	Non-reducing polyketide synthase AusA
470,837	**C**	*A*	T=>K	Moderate	147	*ldc*C	Constitutive lysine decarboxylase
738,146	**A**	*G*					
849,992	**T**	*C*	Syn	Low	148	ACDZ07_04110	MetQ/NlpA family ABC transporter substrate-binding protein
1,051,023	**G**	*GATTCAT*	Insertion	Moderate	36	ACDZ07_05200	GNAT family *N*-acetyltransferase
1,248,573	**T**	*C*	T=>A	Moderate	269	*yfh*O	Lipoteichoic acid-specific glycosyltransferase YfhO
1,413,986	**A**	*G*	Syn	Low	56	*pst*C	Phosphate ABC transporter permease subunit PstC
1,542,411	**C**	*T*	C=>Y	Moderate	263	ACDZ07_07415	Helix-turn-helix domain-containing protein
1,557,108	**C**	*A*	W=>L	Moderate	117	*xer*D	Site-specific tyrosine recombinase XerD
1,682,364	**C**	*T*	Syn	Low	184	*asp*S	Aspartyl-tRNA synthetase
2,201,553	**A**	*G*					
2,556,045	**T**	*G*	Y=>D	Moderate	531	*man*B	Phosphomannomutase
2,577,011	**C**	*T*	V=>I	Moderate	91	*gnt*K	Putative gluconokinase
2,722,031	**G**	*A*	L=>F	Moderate	92	*isa*B	Immunodominant antigen B

*Emboldened and italicized nucleotides are specific to the outgroup of methicillin-susceptible AK3 genomes and genomes within the methicillin-susceptible and methicillin-resistant AK3 lineage, respectively.

†Consequence of SNV relative to ERR7162676 (outgroup). Synonymous change (Syn); non-synonymous changes to protein-coding genes are shown by single-letter amino acid abbreviation (ERR7162676 sequence on the left and SNV impact on the right); blank lines indicate variant in the intergenic region.

**Table 3.** Clade-specific variants for the MRSA AK3 lineage

Position in sa230905_barcode06	sa230905_barcode06 (Reference)*	NZAK3 (branch)*	Change†	Impact	Codon	Gene	Product
99,286	**T**	*C*	L=>S	Moderate	337	*tet*(38)	Tetracycline efflux MFS transporter Tet(38)
237,606	**G**	*T*	V=>F	Moderate	243	ACDZ07_01005	Zinc-binding dehydrogenase
268,432	**A**	*G*	I=>T	Moderate	81	ACDZ07_01155	ABC transporter ATP-binding protein
297,655	**C**	*A*	Syn	Low	156	ACDZ07_01300	5′-Nucleotidase, lipoprotein e(P4) family
541,577	**C**	*T*	Syn	Low	164	*rpl*J	50S ribosomal protein L10
643,271	**A**	*G*					
728,512	**T**	*C*	Syn	Low	202	ACDZ07_03475	Biotin-dependent carboxyltransferase family protein
1,032,205	**C**	*A*					
1,067,855	**C**	*T*	A=>V	Moderate	177	*pur*F	Amidophosphoribosyltransferase
1,167,367	**A**	*C*	L=>F	Moderate	88	arcC1	Carbamate kinase
1,193,138	**A**	*T*	Syn	Low	522	*ile*S	Isoleucine--tRNA ligase
1,217,440	**A**	*G*					
1,435,784	**G**	*C*	I=>M	Moderate	193	ACDZ07_07035	AAA family ATPase
1,465,812	**ATTGTTGTTTTGC**	*A*	Deletion	Moderate	8150	*ebh*	Hyperosmolarity resistance protein Ebh
1,680,288	**T**	*G*	E=>A	Moderate	134	ACDZ07_08145	ThiF family adenylyltransferase
1,751,961	**T**	*C*	E=>G	Moderate	252	*acc*D	Acetyl-CoA carboxylase, carboxyltransferase subunit beta
1,800,235	**A**	*T*	D=>E	Moderate	251	ACDZ07_08700	Hypothetical protein
1,837,199	**G**	*A*					
2,122,507	**C**	*T*	Syn	Low	276	*tex*	Tex family protein
2,144,733	**C**	*T*	G=>E	Moderate	65	*mur*F	UDP-*N*-acetylmuramoyl-tripeptide--d-alanyl-d-alanine ligase
2,266,325	**G**	*A*	H=>Y	Moderate	271	*lac*C	Tagatose-6-phosphate kinase
2,310,728	**C**	*T*	Syn	Low	505	*top*B	DNA topoisomerase III
2,537,681	**C**	*T*	V=>I	Moderate	42	ACDZ07_12735	SDR family oxidoreductase
2,777,570	**C**	*T*					

*Emboldened and italicized nucleotides are specific to the methicillin-susceptible AK3 genomes and genomes within the methicillin-resistant AK3 lineage, respectively.

†Consequence of SNV relative to methicillin-susceptible AK3 genome sa230905_barcode06 (reference). Synonymous change (Syn); non-synonymous changes to protein-coding genes are shown by single-letter amino acid abbreviation (sa230905_barcode06 sequence on the left and SNV impact on the right); blank lines indicate variant in the intergenic region.

To explore the possible origin and distribution of this SaPITokyo12571-like element, we performed a blastn search and a subsequent fast minimum evolution distance tree (built into the NCBI blastn platform) of the complete island sequence from sa230905_barcode06 (GenBank: CP168012) against the NCBI database. Forty-four genomes were identified with >99.9% nucleotide identity and ≥95% query coverage. These genomes were predominantly from human sources across diverse geographical regions, including New Zealand, Australia, Germany, the USA, Japan and Sweden (Fig. S1). One genome originated from a bovine milk sample (Sweden, 2016, GenBank: MN450305) and another from an environmental sample (USA, 2010, GenBank: CP017685). These findings revealed no clear clustering by host or geography and suggest that the SaPITokyo12571-like element is widespread in human *S. aureus* isolates globally, with no strong evidence supporting a livestock- or environment-derived origin based on currently available GenBank metadata.

The AK3 sub-lineage includes 361 of the 397 genomes in this maximum-parsimony phylogeny, most of which were isolated from human samples (*n*=360). The remaining genome represents a strain isolated from bovine milk from a sub-clinical mastitis case in 2015 (SRA: SRR31769156). Most AK3 genomes are from New Zealand (*n*=304), with smaller numbers from Australia (*n*=49), the Netherlands (*n*=4), the UK (*n*=2), Denmark (*n*=1) and Samoa (*n*=1). The high consistency index (0.99) indicates minimal homoplasy in the phylogeny, suggesting reliable reconstruction of evolutionary relationships. The genetic determinants shown in the outer rings demonstrate a consistent and conserved genotypic resistance to methicillin (encoded by *mec*A) and fusidic acid (encoded by *fus*C) within the AK3 sub-lineage. The *in silico spa* typing results reveal t002 as the predominant *spa* type (*n*=231), though other types (present in more than five genomes) including t045 (*n*=6), t062 (*n*=5) and t088 (*n*=5) are also present (Table S5). Within the AK3 lineage, the maximum-parsimony phylogeny reveals three distinct clades (labelled AK3 Clade 1, Clade 2.1 and Clade 2.2). Clade 2.2 contains the highest number of genomes (*n*=186), representing human-derived isolates from New Zealand (*n*=156), Australia (*n*=28), Denmark (*n*=1) and Samoa (*n*=1). Clade 2.1 also includes a substantial number of genomes (*n*=159), with isolates from New Zealand (*n*=136), Australia (*n*=17), the Netherlands (*n*=4) and the UK (*n*=2). Clade 1 is less common, comprising genomes from New Zealand (*n*=12) and Australia (*n*=4). We summarized the distribution of *spa* types and countries of isolation across each clade (Table S7).

### Temporal analysis of ST5 AK3 identifies major divergence dates

This study investigated the evolutionary timeline of the *S. aureus* AK3 lineage from New Zealand. Before using the more computationally intensive BEAST2, we evaluated the temporal signal in the dataset by creating a maximum-likelihood phylogeny with TempEst. The sequence data for a publicly available genome (SRA: SRR15602515) and sample 22MR0788 (SRA: SRR31718605) were identified as outliers (possibly indicating an error in the sequence data or sample metadata) and so were excluded from the root-to-tip regression (Fig. S2a). The AK3 lineage demonstrated a linear relationship (correlation coefficient=0.57) between divergence time and evolutionary distance (Fig. S2b). Based on the maximum-likelihood phylogeny, the estimated mutation rate was 2.42×10^−4^ substitutions per site per year (*R*^2^=0.33); however, this rate does not represent a genome-wide mutation rate due to the use of a core-genome SNV alignment that excludes invariable sites. This exploratory analysis suggests clock-like behaviour in the dataset, with the most recent common ancestor (MRCA) to the AK3 lineage estimated to have emerged around 1974 (95 % confidence interval: 1966 to 1980).

**Fig. 5.**
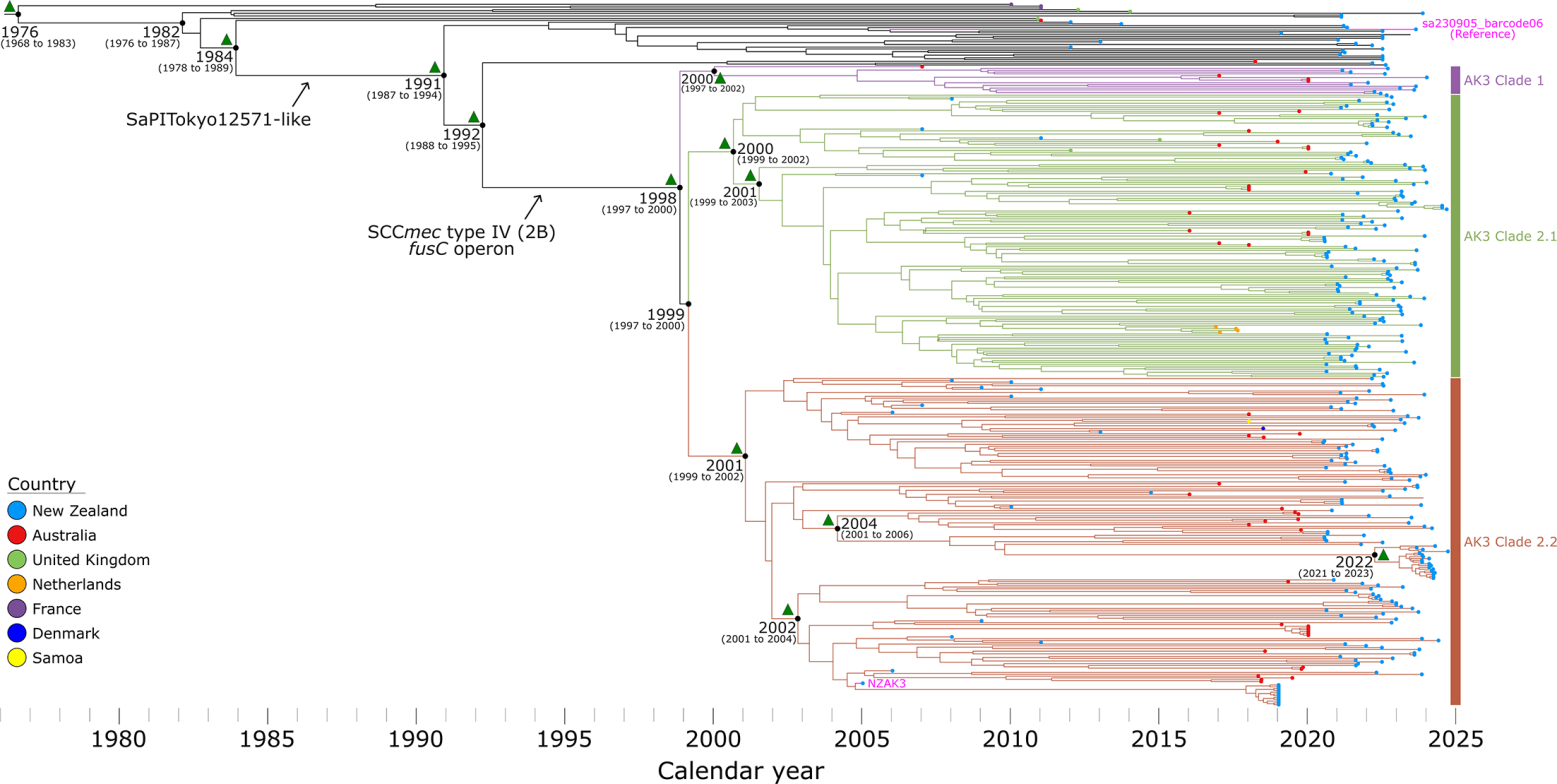
Evolutionary reconstruction of the *S. aureus* AK3 lineage. A time-calibrated maximum clade credibility tree was inferred from 9,801 non-recombinant orthologous biallelic core-genome SNVs from 395 genomes. SNVs were derived from a core-genome alignment of ~2,406,900 bp and were called against the chromosome of sa230905_barcode06 (GenBank: CP168012). SNV density filtering in SPANDx (excluded regions with three or more SNVs in a 10 bp window). The x-axis represents the emergence time estimates. The green triangles represent nodes with posterior probabilities greater than 0.95.

Once we confirmed the presence of a temporal signal, we employed the nested sampling Bayesian computation algorithm to determine the best-fitting tree model and generate a time-calibrated phylogeny. The results of the nested sampling algorithm favoured the relaxed clock model. The Bayesian skyline population size change model was selected, with a marginal likelihood estimate of −89,807.20 (sd: ±20.38) (Table S8). Using BEAST2 (median node heights), the estimated time to the MRCA of the AK3 lineage was ~1976, with a 95% highest posterior density (HPD) interval from 1968 to 1983 (Fig. 5). The median evolutionary rate calculated by BEAST2 is 2.59×10^−4^ substitutions per site per year (95% HPD: 2.41×10^−4^ to 2.77×10^−4^), consistent with the rate observed in root-to-tip divergence analysis (Fig. S2b). To adjust for ascertainment bias, the dataset describes one SNV for every 245.6 bases across the ~2,406,900 bp core genome, resulting in a genome-wide mutation rate of 1.06×10^−6^ (95% HPD: 0.98×10^−6^ to 1.13×10^−6^) mutations per site per year, aligning with previous studies [42,80,82]. This corresponds to 2.5 (95 % HPD: 2.4 to 2.7) fixated SNVs per year per genome, meaning isolates sharing an MRCA 1 year prior would typically differ by four to six SNVs. To validate this estimate, we performed a confirmatory analysis by explicitly incorporating the number of invariant sites into the BEAST2 XML file (i.e. A=939,412; C=455,550; G=461,101; T=941,291). This approach yielded a slightly lower median genome-wide mutation rate of 8.85×10⁻⁷ (95% HPD: 8.25×10⁻⁷ to 9.46×10⁻⁷), demonstrating that while the estimate is sensitive to the assumed number of invariant sites, the values remain within a similar range (Fig. S3), supporting the robustness of our calculation.

The evolutionary timeline revealed that the *S. aureus* AK3 lineage likely originated in Europe, as evidenced by genomes representing MSSA isolates from France (*n*=3) and the UK (*n*=2) positioned at the basal portion of the tree (Fig. 5). The MSSA ancestor of the current MRSA AK3 lineage was likely introduced to New Zealand in the late 1970s or 1980s, prior to the acquisition of the SaPITokyo12571-like genomic element (Figs 3 and S4), which carries SEC bovine variant (*sec*-bov) and an enterotoxin-like protein (*sel*). A crucial evolutionary event occurred around 1998 (95% HPD: 1997 to 2000) when the MSSA lineage acquired the SCC*mec* type IV (2B) element and adjacent *fus*C operon (Fig. 4). This acquisition led to the emergence of three distinct clades: AK3 Clades 1, 2.1 and 2.2. The presence of isolates from New Zealand and Australia throughout all major clades indicates extensive regional transmission between these countries. The identification of isolates from the Netherlands, Denmark and Samoa demonstrates the subsequent international dissemination of these lineages.

### Genomic analysis of a bovine-derived isolate highlights gaps in sampling

**Fig. 6.**
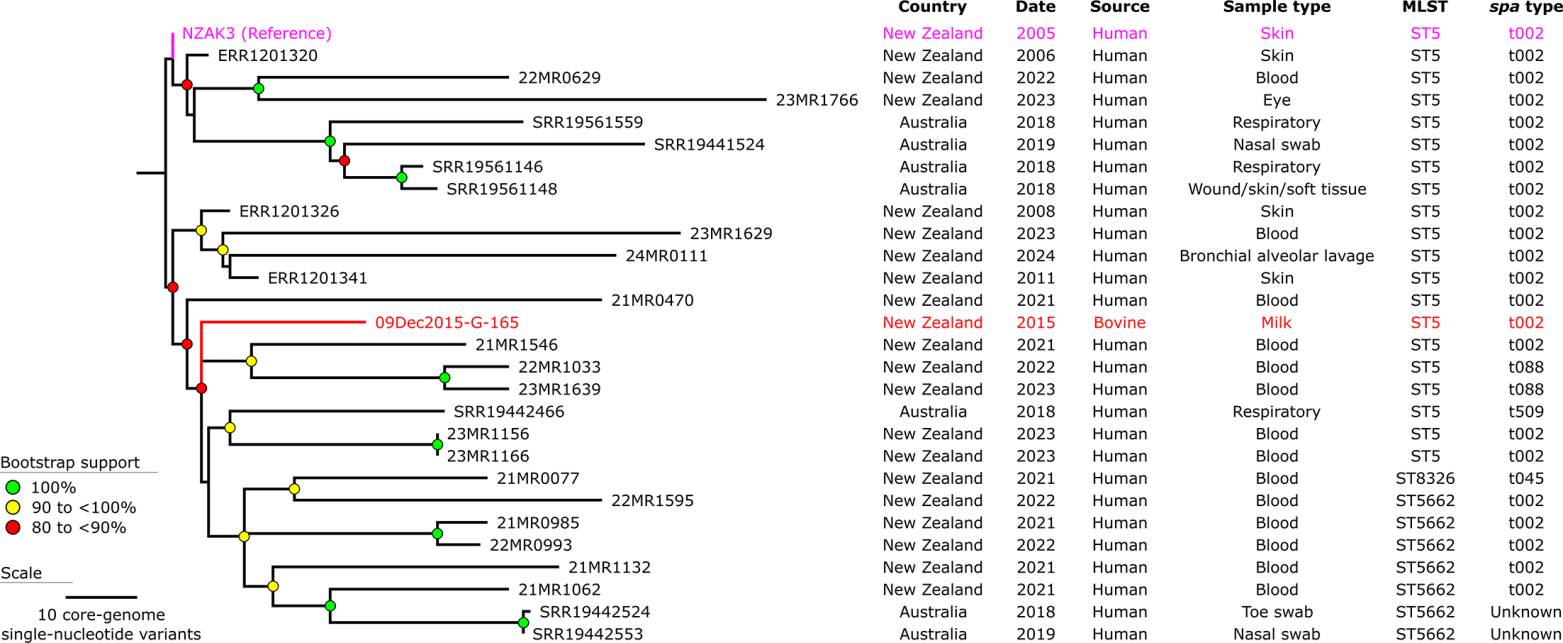
Maximum-parsimony phylogeny of a subset of the *S. aureus* AK3 Clade 2.2 lineage. The phylogeny was inferred from 819 non-recombinant orthologous biallelic core-genome SNVs from 28 genomes. SNVs were derived from a core-genome alignment of ~2,699,000 bp and were called against the chromosome of NZAK3 (GenBank: LT009690). The consistency index for the tree was 1.0. SNV density filtering in SPANDx (excluded regions with three or more SNVs in a 10 bp window). The phylogenetic tree was rooted according to the MSSA ST5 SRR19441781 outgroup (collected in Australia), which has been omitted for visualization.

Most (*n*=360) of the 397 genomes represent *S. aureus* genomes isolated from human samples. A single genome representing strain 09Dec2015-G-165 (SRA: SRR31769156) was isolated from bovine milk in 2015. To investigate the relationship of this bovine-derived genome within Clade 2.2 of the AK3 lineage, we focused on a subset (*n*=28) of genomes from this clade (Fig. 6). The phylogenetic tree, constructed using core-genome SNVs, is well-supported, with most nodes having bootstrap values of ≥90%, reflecting high confidence in the inferred relationships. While this single bovine-derived isolate clusters with human-derived isolates in the AK3 lineage (Clade 2.2), strain 09Dec2015-G-165 is genomically distinct (Fig. 6). The genome of 09Dec2015-G-165 is separated by 60 pairwise SNVs from genome 21MR1546 (SRA: SRR31718884), a human-derived blood isolate collected in December 2021. This degree of divergence might suggest a potential zoonotic link; however, it more likely reflects substantial gaps in sampling data, underscoring the need for enhanced surveillance to clarify the evolutionary and transmission dynamics of contemporary circulating lineages.

## Discussion

This research expands the genomic foundations and evolutionary history of the ST5-MRSA-SCC*mec*-IV (or AK3 strain), which is a prolific contributor to MRSA infections in New Zealand [20]. By conducting a phylogenetic analysis of 397 *S*. *aureus* AK3-associated genomes, including 285 recently sequenced genomes, we identified key genomic factors and evolutionary events that have likely driven the expansion of the AK3 lineage. The predominance of *spa* type t002 among AK3 genomes is a valuable epidemiological marker for tracking AK3-related infections [5]. Although AK3 appears to represent a primarily localized expansion (Figs2  5), its detection in Australia and Europe raises concerns about potential broader dissemination, warranting continued monitoring [2,15, 18].

Compared with earlier efforts on the AK3 strain in New Zealand [2,3, 15, 18], which laid the groundwork by characterizing its introduction, spread and genomic features, this study extends understanding by analysing a broader temporal range (spanning an additional 20 years) and incorporating national sampling from multiple hospitals and regions, thereby improving representation of the AK3 lineage across New Zealand. Additionally, we identified mobile genetic elements and accessory genes potentially contributing to the persistence and resistance of AK3-related strains, addressing gaps in the previous understanding of its genetic drivers. Although only a single bovine isolate was identified within the AK3 clade, its presence raises the possibility that non-clinical reservoirs could contribute to the lineage’s ecology, an aspect not explored in prior research [2,15]. However, in a recent survey of 218 farm ruminant isolates, only one methicillin-susceptible CC5 isolate was identified, and it carried ruminant virulome [83]. This finding, alongside existing evidence of MRSA in animal hosts, supports the need for further investigation into the role of non-clinical reservoirs in AK3’s persistence and spread. While we identified key genetic factors associated with AK3 dominance, functional studies are needed to elucidate their precise roles in fitness, virulence and transmissibility.

### Genomic adaptations driving AK3 success

Our findings suggest that the success of AK3 stems from a combination of genomic adaptations that may have enhanced resistance to antibiotics and transmissibility. The stepwise acquisition of mobile genetic elements, first the SaPITokyo12571-like element [84], followed by SCC*mec* type IV (2B) encoding the *fus*C operon, has been pivotal in shaping the evolutionary success of AK3 (Fig. 5). The acquisition of SCC*mec* type IV (2B) and the *fus*C operon, which confer resistance to methicillin (via *mec*A) and fusidic acid (via *fus*C), respectively, aligns with the observed increase in fusidic acid usage in New Zealand [2,15, 18]. The stable retention of these elements within AK3 (Fig. 2) underscores their selective advantage under antibiotic pressure.

The SaPITokyo12571-like element identified in AK3 and the basal MSSA lineage carries the SEC bovine variant (*sec*-bov) and an enterotoxin-like protein (*sel*) (Fig. 3), both of which are associated with pathogenicity (by modulating the bovine immune response) in *S. aureus* [85]. The SEC bovine variant, a form of enterotoxin C found in bovine strains, may have distinct antigenic properties compared with human-associated SEC, possibly contributing to differences in virulence [85,88]. The enterotoxin-like protein (*sel*) may contribute to virulence by triggering immune responses or disrupting normal cellular functions [89]. These findings emphasize the need for a more thorough sampling of bovine sources to fully capture the diversity of *S. aureus* strains and gain a better understanding of their potential for transmission and pathogenicity. This gap in sampling limits our understanding of the interplay between host-specific adaptations and how *S. aureus* evolves and adapts in different environments, such as cattle farms, where these strains could significantly impact both animal and public health [24].

### Sampling limitations and implications

A key strength of this study is the integration of newly sequenced genomes with publicly available data, enabling a comprehensive and robust analysis of the AK3 lineage. However, our reliance on publicly available genomes introduces potential biases due to the underrepresentation of certain geographic regions. Similarly, there is likely a geographic bias in the isolates sequenced for this study, as most genomes analysed are from urban centres (hospitals), with possible limited representation from rural or remote areas of New Zealand. Despite the comprehensive genomic dataset, our study highlights a critical gap in the sampling strategy. Of the 397 genomes analysed, only two MSSA genomes (both outside of the AK3 lineage) were derived from bulk milk tanks (strain AB93, SRA: SRR29758833; strain AB94, SRA: SRR29758603). Only one MRSA AK3-related genome (strain: 09Dec2015-G-165) was cultured from bovine milk in 2015 [22]. Despite the large sample size from human sources, this limited representation of bovine sources underscores a significant under-sampling of bovine reservoirs of *S. aureus*. The substantial genomic diversity observed across the AK3 lineage (Fig. 2), coupled with limited sampling from potential reservoirs (e.g. community, environment or animal sources), suggests that additional strain variants may remain undiscovered in New Zealand. Fusidic acid, a key resistance determinant within the AK3 lineage, is commonly prescribed in veterinary medicine [90], particularly for dogs and cats, where it is primarily used topically to treat skin and eye infections caused by Gram-positive bacteria, including *Staphylococcus* spp. Broader sampling of bovine, other animal and environmental sources is essential to fully capture the diversity and evolutionary dynamics of *S. aureus* in New Zealand. Such efforts could also inform strategies to mitigate transmission risks between human and animal populations [24].

### Public health implications

The predominance of AK3 among New Zealand MRSA isolates [20] and the associated resistance to fusidic acid (an antibiotic that has been widely used in topical formulation) poses significant challenges for infection control. The clade-specific SNVs identified in the branch associated with SaPITokyo12571-like element acquisition (MSSA ancestor) (Table 2) and subsequent MRSA AK3 emergence (Table 3) may influence fitness, virulence and/or transmission potential, although further experimental and phenotypic validation is required. The geographic clustering of AK3, particularly its high representation in New Zealand, underscores the critical role of regional genomic surveillance in tracking the emergence and spread of CA-MRSA lineages. Furthermore, the disproportionate burden of MRSA infections among vulnerable populations, including Māori and Pacific peoples, emphasizes the need for culturally responsive and equitable public health interventions [20]. These findings underscore the importance of integrating genomic surveillance with community-level health strategies to address disparities in infection control outcomes.

### Broader One Health context

One Health emphasizes the interconnectedness of human, animal and environmental health, particularly in the context of AMR [91,93]. While significant progress has been made, under-sampled populations such as the Māori and Pacific peoples, environmental reservoirs and bovine herds remain critical gaps in AMR research. Limited environmental sampling, especially in potential unknown sinks, further constrains our understanding of how resistance genes disseminate and persist in different ecological niches. Expanding research efforts to include these populations and potential reservoirs will provide a more comprehensive view of the AMR network, informing targeted mitigation strategies.

### Concluding remarks

In conclusion, this research advances our understanding of the evolutionary and epidemiological dynamics of the MRSA AK3 lineage. By linking genomic adaptations to environmental and clinical pressures, our findings demonstrate mechanisms underlying the success of AK3 and may inform strategies for its control. However, addressing current gaps in sampling and performing functional studies to validate key genetic factors will be essential to fully understand the fitness, virulence and transmissibility of the AK3 strain. Continued genomic surveillance, particularly in underrepresented reservoirs, remains critical for mitigating the public health impact of AK3 and enhancing global efforts to combat CA-MRSA.

## Supplementary material

10.1099/mgen.0.001452 Uncited Supplementary Material 1.

## Abbreviations

AMR: antimicrobial resistance
BAPS: Bayesian analysis of population structure
bp: base pair
CA-MRSA: community-associated methicillin-resistant *Staphylococcus aureus*
CC: clonal complex
ESR: Institute of Environmental Science and Research
EUCAST: European Committee on Antimicrobial Susceptibility Testing
GTR: general time-reversible
HERRO: haplotype-aware error correction
HPC: High-Performance Compute
HPD: highest posterior density
IPC: infection prevention and control
LIC: Livestock Improvement Corporation
MBIE: Ministry of Business Innovation and Employment
MLST: multi-locus sequence typing
MPI: Ministry of Primary Industries
MRCA: most recent common ancestor
MRSA: methicillin-resistant *Staphylococcus aureus*
MSSA: methicillin-susceptible *Staphylococcus aureus*
NCBI: National Center for Biotechnology Information
NICU: Neonatal intensive care unit
ONT: Oxford Nanopore Technologies
ORF: open reading frame
PBS: Phosphate-buffered saline
PCR: Polymerase chain reaction
PFGE: Pulsed-field gel electrophoresis
PVL: Panton–Valentine leukocidin
SAB: *Staphylococcus aureus* bacteraemia
SCC*mec*: staphylococcal cassette chromosome *mec*
SD: standard deviation
*sec*-bov: staphylococcal enterotoxin C bovine variant
*sel*: enterotoxin-like protein
SNV: single-nt variant
*spa*: staphylococcal protein A gene
SRA: Sequence read archive
ST: sequence type
WGS: whole-genome sequencing
WSPP: Western Samoan phage pattern
